# Clinical Communications

**Published:** 1836-07

**Authors:** 


Art. II.—*Klinische Mittheilungen, von* Dr. F. A. G. Berndt, Königl. Geheimen Medizinal-Rathe, u. s. f.—*Greifswald*, 1833-4. Heft I. II. *Clinical Communications, by* Dr. F. A. G. Berndt.—*Greifswald.* Part I. 1833. 8vo. pp. 166. Part II. 1834. 8vo. pp. 195.

Dr. Berndt is the director of the medical and obstetrical *clinic* in the university of Greifswald, and has embodied a part of his experience at that institution in the volumes now before us. We recommend the work to our readers who are German scholars, as a valuable one: we can only find room for a brief analysis of some of the more practical articles contained in it.

The clinical institution at Greifswald consists of a small hospital, with out as well as in patients, many of whom are visited at their own homes, so that it combines what we should call hospital and dispensary practice.

The number of patients treated in it, from 1824 to 1833, was 6,226; of whom, 975 were cases of ague, 613 of bilious fever, and 435 are set down under the head of *status gastrici congestivi.* It is almost a truism to remark that, in summaries of this kind, the numbers set down to the several diseases vary more in consequence of the prevalence of different medical theories, or the unequal diligence of physicians in investigating some forms of disease, than from the morbific influence of climate on habits: thus, in the present registers, we find 266 cases of pneumonia and 116 of bronchitis, but only four of organic disease of the heart. As an instance on the other side of the question, however, we may mention ten cases of poisoning by cheese; an accident constantly recorded by German writers, but quite unknown in Britain.

*Intermittent Fever.* In the chapter on this subject, Dr. Berndt attempts to determine the best method of curing intermittents, and the smallest doses of cinchona or its preparations required for that purpose. He was especially led to the latter investigation by the smallness of the funds placed at his disposal, and tried the two following methods:

1. The one recommended by Trassoni, Torti, Cullen, Thuessins, Mursinna, Nasse, and others, of giving cinchona or quinine a short time before the paroxysm.

2. The method of giving small doses of these remedies during the paroxysm itself.

He gives a table containing the results of fifty-five cases treated by administering a scruple of cinchona an hour before the paroxysms. Of these, thirty were tertians; of which, twenty-three were cured without any return of the paroxysm, and six after one return; one case was not cured. Nineteen were quotidians; of which, four were cured without any return of the paroxysm, and thirteen after one return; two cases were not cured. Six were quartans; and this method failed in all.

This method was equally successful at all times, excepting that, in the year 1829, relapses were common after this, as well as after every other variety of treatment. It is to be remarked, that a second dose was always given before the expected time of the second paroxysm, so that on the whole two scruples of cinchona were employed.

A second table gives the results of twenty-four cases treated with a single dose of quinine, administered an hour before the paroxysm. Fourteen of these were tertians, and ten quotidians; and, with the exception of one tertian, they were all cured by a single dose; but in every case one paroxysm occurred after the remedy had been taken, so that in this respect the results were not quite so favorable as those of the former table.

A third table gives the results of thirty-five cases treated with a scruple of cinchona three hours before the paroxysm, another scruple two hours before, and another scruple one hour before. The fevers were all quotidian or tertian, one of the latter being a double tertian in a child five years old. The treatment was successful in every case: in sixteen, the fever was immediately checked; in nineteen, it returned once. It may be worth remarking that our author, whose experience in the treatment of intermittents is ample, does not scruple to give these large doses of cinchona to children of four, five, seven, and eight years of age. The patients in this table did not require the set of doses to be repeated: this has occasionally been necessary in our author's practice, but rarely.

The next table contains the results of thirty-nine quotidians and tertians, treated with a scruple of cinchona at the commencement of the paroxysm, a scruple during the middle, and a scruple at the termination. The youngest patient in this table was eight years old, but there were several of twelve, thirteen, and fourteen. The first dose, which was given during the rigor, was often rejected by vomiting, but without any influence on the success of the treatment, as every case was cured without a single return of the paroxysm, nor was any other ill effect produced. This method was only adopted when an emetic had been previously exhibited, and the case was free from complication.

Among nearly a thousand intermittents, only forty-four were of the quartan type: these, however, were very obstinate cases, not only from the well-known severity of this form of ague, but from many of them being cases of long standing, sent to Greifswald from different parts of the province. Dr. Berndt endeavours to cure them without cinchona, believing that this remedy has a tendency to produce the secondary diseases which so often follow quartan ague. His favorite remedy is extract of hellebore, in combination with muriate of ammonia and bitters; and he affirms that, with this treatment, the ague disappears after the third or fourth paroxysm, the cachexia and other bad symptoms disappearing at the same time, without the use of bark. In some few cases this does not take place, and the best thing is to administer the preparations of cinchona with belladonna, as well as the combination just mentioned. The following is our author's favorite prescription:

℞. Extr. Hellebori,

Ammon. Muriat. āā ʒij.

Ext. Absinthii, ʒj.

Solve in Aquæ Menth. Pip. ℥v. sit dosis coch. j. max. 2ª q. horâ.

A table of thirteen cases is added, which shows the great value of this remedy in quartan ague.

There was an interesting case of a double quartan occurring in a boy of sixteen, who had already been labouring three years under intermittent fever. One of the fevers was soon cured by the hellebore, but the second was obstinate, and required to be treated with bark and belladonna.

The endermic method was frequently tried, particularly in children; and it was found that intermittents could generally, though not always, be cured by sprinkling quinine on the sore caused by a small blister. The ulceration produced by the drug, however, is so troublesome as to form a strong objection to this mode of treatment.

Salicine, piperine, and prussiate of iron were also tried, and with the following results:

(1.) Twenty-two cases were treated with the prussiate of iron; and, of these, twelve were perfectly cured in three or four days, the patients taking from four to six grains three or four times a day: in ten cases the remedy had no effect.

(2.) Eight cases were treated with piperine, of whom three only were cured; in the other five, the continued exhibition of the remedy had no effect.

(3.) The salicine was given to five patients, in doses of from two to four grains every two hours during the absence of the fever, in tertians, but without any effect.

*Scarlatina.* The fourth paper in the second volume is on the malignant forms of scarlet fever, with reference to the variety of the cerebral affections accompanying this disease. The practical point upon which Dr. Berndt insists is, that, while scarlet fever may be accompanied by inflammation of the brain, to be treated by bleeding, it may also be accompanied by a specific affection of the brain not depending on inflammation, far less on congestion, as Armstrong would have us suppose. This sort of *narcotism* produced by scarlatina, (*Scharlach-Toxication,*) is chiefly distinguished by the absence of vomiting; the suddenness, great extent, and dark colour of the eruption; the frequent, soft, and often irregular pulse; restlessness; disturbance of the brain. Our author remarks, that chlorine in the south of Germany, and carbonate of ammonia in the north, have been vaunted as infallible specifics against scarlatina, but he had tried them both without profit: for scarlatina, when mild, gets well of itself; when severe, bids defiance to the specifics.

*Hooping-cough.* The fifth paper is on the efficacy of the acetate of morphia, applied in the endermic method, in hooping-cough.

Dr. Berndt seems to have tried most of the remedies which have any repute in the treatment of hooping-cough. He has often given belladonna in different methods, in large doses, and for a long time. In most cases it did nothing, in many its utility was dubious, in a few it diminished the number and violence of the paroxysms. He has made extended experiments with prussic acid, but has seen its use attended with advantage only in recent cases; for, in fully developed hooping-cough, it had no good effect. Nor has he derived any advantage from hyoscyamus, aqua lauro-cerasi, digitalis, stramonium, or conium. Opium, however, has been beneficial towards the end of the disease, when the mucous stage has been protracted. He has tried assafœtida, the preparations of ammonia, musk, valerian, muriatic acid, tincture of cantharides, oxide of zinc, ammoniated copper, and acetate of lead; and is convinced that they are all useful under some circumstances, but have no especial power to cure pertussis. Autenrieth's method, which consists in rubbing-in tartar-emetic ointment, rarely produces an advantage equivalent to the torment inflicted on the child.

Strict antiphlogistic measures, the detraction of blood and administration of calomel, are not more efficacious.

Emetics are superior to any of these remedies, when exhibited with due attention to all existing circumstances.

Dissatisfied, however, with all these medicines, Dr. Berndt determined to try the acetate of morphia in the endermic method, and he details sixteen cases in which it was employed: we will give the first at length.

A stout girl, æt. 8, had suffered from hooping-cough for four weeks, for which she had been treated without advantage. The disease had reached the acme of the convulsive stage, (a violent paroxysm occurring almost every half-hour,) but was free from complication, excepting that blood was sometimes coughed up. It was resolved to exhibit the acetate of morphia endermically, and accordingly a blister, of the size of an eight-groschen piece, (a shilling,) was placed upon the epigastrium. When it had drawn, one grain of acetate of morphia and four of gum arabic were sprinkled upon the spot, and a bit of dry rag was put over it. This was repeated on the 30th of March, and on the 1st, 2d, and 3d of April. Amendment took place, but not very decidedly. On the 4th of April, therefore, a fresh blister was put on, and the dose of morphia was increased on the 5th to a grain and a half. In the course of a few hours symptoms of narcotism came on. The child's face became red and puffed up, the head was heavy and confused, and a state of perfect sopor followed, which lasted more than twelve hours. A profuse perspiration then broke out, and the sopor gave way; but the child still complained of pain, heaviness, and confusion of the head. During the sopor the child had coughed but twice, which had not roused her. The following night she slept soundly, and coughed slightly five times; so that from this time the number of paroxysms was considerably diminished, and their violence had almost entirely disappeared. One grain of the acetate was now sprinkled on the sore every forty-eight hours; and on the 12th of April the hooping-cough was so far quelled that there were only about two paroxysms every twenty-four hours. The fits, too, had entirely lost their convulsive character, so that it was impossible to gainsay the efficacy of the acetate of morphia, though it was not so very striking as some physicians have made it out.

The general result was that, of the sixteen patients, six were attacked with narcotism, and these alone experienced much benefit from the morphia. In ten cases the effects of the remedy were not pushed so far, and of these four obtained a mitigation of the paroxysms, but not a cure. The other six patients derived no benefit from the medicine. Hence it appears that it is certainly possible to relieve the convulsive stage of hooping-cough by morphia pushed till it produces narcotism; but we must pause, says our author, before we can recommend so bold and dangerous a proceeding.

*Diabetes* The last and longest paper in the volume contains the history of six cases of diabetes mellitus, for the details of which we must refer to the work itself. Two of the cases were permanently relieved; three of the patients were carried off by attacks of acute diseases unconnected with the diabetes, and one sunk under colliquative symptoms after the original affection had nearly disappeared.

The post-mortem appearances in the four fatal cases were not very remarkable. The kidneys were never found diseased, and the parietes of the bladder were thickened in one case only. In two cases the stomach was immensely enlarged, a natural consequence of the enormous quantity of food and drink swallowed.

Dr. Berndt concludes with some observations on the various remedies proposed for the cure of diabetes; we must content ourselves with a few of them.

*Emetics* are excellent palliatives.

*Opium* diminishes the hunger, thirst, and quantity of urine; acetate of morphia is still better, as it does not constipate, yet it is unable to cure the disease.

*Ammoniated copper* checks the formation of sugar, and acts favorably upon the nerves of the stomach. It may be given in this disease in very large doses, such as from ten to sixteen grains a day.

*Animal charcoal* improves the quality of the urine a little.

*Creosote* improves the quality of the urine and diminishes its quantity.

Our author has found, like Rollo and the majority of physicians, that a meat diet is the best. Camphor, alum, phosphorus, balsamic remedies, iodine, bitters, (as aloes, ox-gall,. and quassia,) phosphoric acid, sulphuric acid, solution of chlorine, sulphuret of potass, arsenic, mercurial inunction, absorbents, carbonate of ammonia, ethereal animal oil, inunction of oil, baths, carbonate of iron, and iron filings, were all useless.

